# An Analysis of COVID-19 Mortality in Chhattisgarh: A Two-Year Study on Demographics, Trends, and Impacts

**DOI:** 10.7759/cureus.43155

**Published:** 2023-08-08

**Authors:** Sanjana Agrawal, Dharmendra K Gahwai, Sonal Dayama, Abhiruchi Galhotra

**Affiliations:** 1 Medicine, State Health Resource Centre, Raipur, IND; 2 Health Services, Government of Chhattisgarh Raipur Directorate of Health Services, Chhattisgarh, IND; 3 Community Medicine, Employees' State Insurance Corporation (ESIC) Medical College Hyderabad, Telangana, IND; 4 School of Public Health, All India Institute of Medical Sciences, Raipur, IND

**Keywords:** india, mortality, chhattisgarh, covid-19, pandemic

## Abstract

Background

The COVID-19 pandemic, caused by the novel coronavirus, has had a profound impact on global health, significantly affecting demographics worldwide. As India's twelfth most affected state, Chhattisgarh has experienced substantial COVID-19-related fatalities. This study aims to analyze the temporal, geographical, and demographic distribution and trends in COVID-19 mortality reported by the Department of Health and Family Welfare, Chhattisgarh, spanning from the onset of the pandemic until March 2022 (two years).

Methods

Data about all COVID-19 deaths recorded between March 2020 and March 2022 were collected from the State Surveillance Unit, Department of Health and Family Welfare, Chhattisgarh, and subsequently compiled in a Microsoft Excel sheet (Microsoft, Redmond, Washinton) for analysis.

Results

A comprehensive dataset of 14,038 deaths was examined during the study period. Of these, 24.5% (3446), 72.2% (10141), and 3.3% (451) occurred in 2020, 2021, and 2022, respectively. The top five districts in Chhattisgarh with the highest COVID-19 mortality rates were identified as follows: 1) Raipur (23.5%), 2) Durg (13.4%), 3) Bilaspur (8.9%), 4) Raigarh (7.05%), and 5) Janjgir Champa (6.25%). The mean age of the deceased individuals was determined to be 55.44 years, with a standard deviation of 15.14 years. Furthermore, the impact of the pandemic was found to affect males compared to females in Chhattisgarh disproportionately.

Conclusion

Over the two-year study period, three distinct waves of COVID-19 were observed, with the second wave being the most devastating, particularly for the elderly population. Understanding the demographic characteristics and trends in COVID-19 mortality is crucial for implementing targeted public health measures and interventions to mitigate the impact of future infectious disease outbreaks.

## Introduction

Throughout history, numerous epidemic diseases, such as cholera, bubonic plague, measles, and influenza, have inflicted devastating tolls on global populations, claiming millions of lives. However, recently, a novel infectious disease, COVID-19, emerged as a formidable global health crisis. Caused by the novel coronavirus, COVID-19 has unleashed catastrophic consequences on demographics worldwide.

The top three countries severely affected by this pandemic are the USA, India (accounting for over one-tenth of the world's confirmed cases), and Brazil, grappling with the most infections and deaths. The impact has been particularly severe in India, with the second-highest number of cases after the USA. The devastating second wave of COVID-19, which occurred between April and June 2021, accounted for more than 50% of all COVID-19 deaths in the country. India's Ministry of Health and Family Welfare reported 235,986 COVID-19 deaths, comprising 56% of the overall death toll since the outbreak [1].

Chhattisgarh, the twelfth-highest affected state in India, has also witnessed a significant mortality toll, reporting 14,034 deaths by 2022 [2]. Notably, the case fatality rate (CFR) in Chhattisgarh stands at 1.22%, the third-highest in India after Maharashtra (1.88%) and Delhi (1.38%), slightly above the national average.

Considering these alarming statistics, this study aims to comprehensively analyze the time, place, and person distribution of all reported COVID-19 deaths by the Department of Health and Family Welfare, Chhattisgarh, from the onset of the pandemic until March 2022, encompassing two years. By delving into the trends and patterns of COVID-19 mortality in the state, this research seeks to provide valuable insights for devising targeted strategies and effective public health interventions to mitigate the impact of the pandemic in this region.

## Materials and methods

Study setting

The study encompassed all districts in the State of Chhattisgarh, including rural, urban, and tribal areas, to ensure a comprehensive representation of the entire population.

Study design

A retrospective cross-sectional record-based study design was employed. This design allows the researchers to examine data for a defined period without altering or intervening during events.

Sampling strategy

The sampling strategy utilized in this study involved collecting all available death records from the State Surveillance Unit, Department of Health and Family Welfare, Chhattisgarh, from March 2020 to March 2022. This approach ensured that the dataset was complete and representative of the entire population during the two-year study period.

Inclusion criteria

All death records available at the State Surveillance Unit within the specified study period were included in the analysis. By having all records, the study sought to minimize selection bias and enhance the accuracy of the findings.

Exclusion criteria

To maintain data quality and integrity, any death records deemed illegible or lacking essential information, such as age and gender, were excluded from the study.

Data collection

Data collection involved obtaining death records from the State Surveillance Unit, Department of Health and Family Welfare, Chhattisgarh. These records were then carefully entered into a Microsoft Excel spreadsheet (Microsoft, Redmond, Washington), ensuring meticulous data handling and minimizing errors.

Data analysis

The collected data underwent a thorough cleaning process to eliminate inconsistencies or errors. Subsequently, the dataset was coded for descriptive analysis to provide a comprehensive overview of the mortality patterns observed during the study period. Statistical measures such as frequency and percentage were used to summarise the data. Furthermore, the data were categorized year-wise, and the Chi-squared test analysis and p-values were calculated using SPSS software (IMB Inc., Armonk, New York) to assess associations and significance levels between variables.

Ethical considerations

Ethical approval was obtained from the institutional review board (All India Institute of Medical Sciences, Raipur, Chhattisgarh, India) to ensure that the study complied with all ethical guidelines and safeguarded the privacy and confidentiality of the deceased individuals' information vide Letter No. 2104/IEC-AIIMSRPR/2021 dated 27/12/2021.

## Results

**Figure 1:**
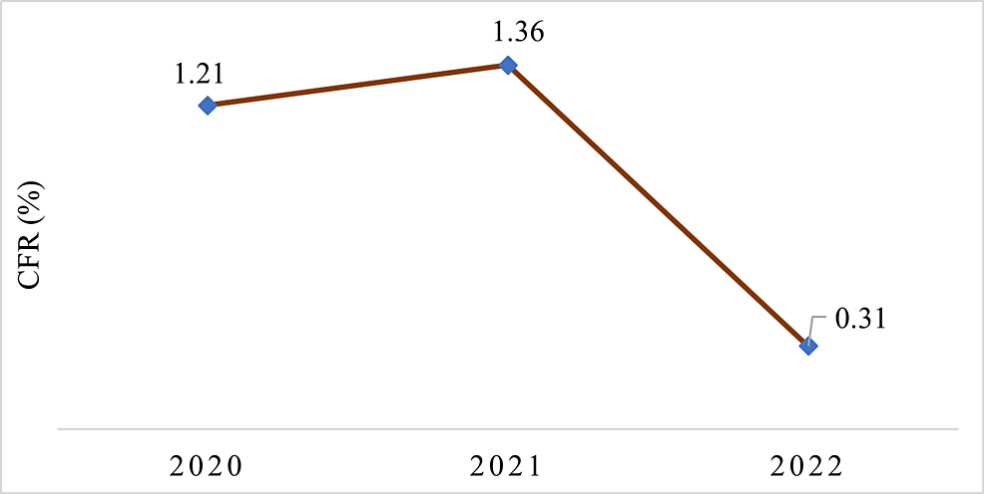
Case fatality rate (CFR)

The study encompassing the period from March 2020 to March 2022 in the State of Chhattisgarh revealed 14,038 deaths attributed to the COVID-19 pandemic. The distribution of deaths across the three years showed that 24.5% (3446) of the deaths occurred in 2020, 72.2% (10141) in 2021, and 3.3% (451) in 2022, indicating a significant toll on human lives throughout the pandemic. Notably, the second year of the pandemic (2021) experienced the highest number of deaths, accounting for more than 70% of the total deaths reported. The case fatality rate (CFR) analysis demonstrated variations across the years, with the state's highest CFR of 1.36% observed in 2021 and the lowest CFR of 0.31% recorded in 2022 (Figure 1). These findings highlight the severity of the pandemic's impact on Chhattisgarh.

**Figure 2:**
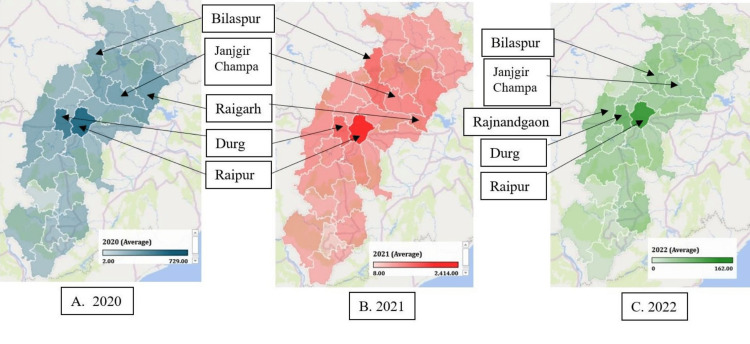
COVID-19 deaths in Chhattisgarh (year-wise)

The study findings revealed the top five districts in Chhattisgarh with the highest COVID-19 mortality rates, with Raipur accounting for 23.5% of the deaths, followed by Durg (13.4%), Bilaspur (8.9%), Raigarh (7.05%), and Janjgir Champa (6.25%). These urban districts collectively contributed to approximately 60% of the total deaths in the state throughout the consecutive years of the pandemic (Figure 2). Interestingly, the central part of Chhattisgarh exhibited the highest number of COVID-19 deaths, while the northern and southern regions contributed comparatively fewer deaths. Remarkably, the tribal districts, including Narayanpur, Sukma, Dantewada, Bijapur, and Kondagaon, reported the least fatalities, accounting for only 1.5% of the total deaths in the state. These findings shed light on the geographical disparities in COVID-19 mortality within Chhattisgarh.

**Figure 3:**
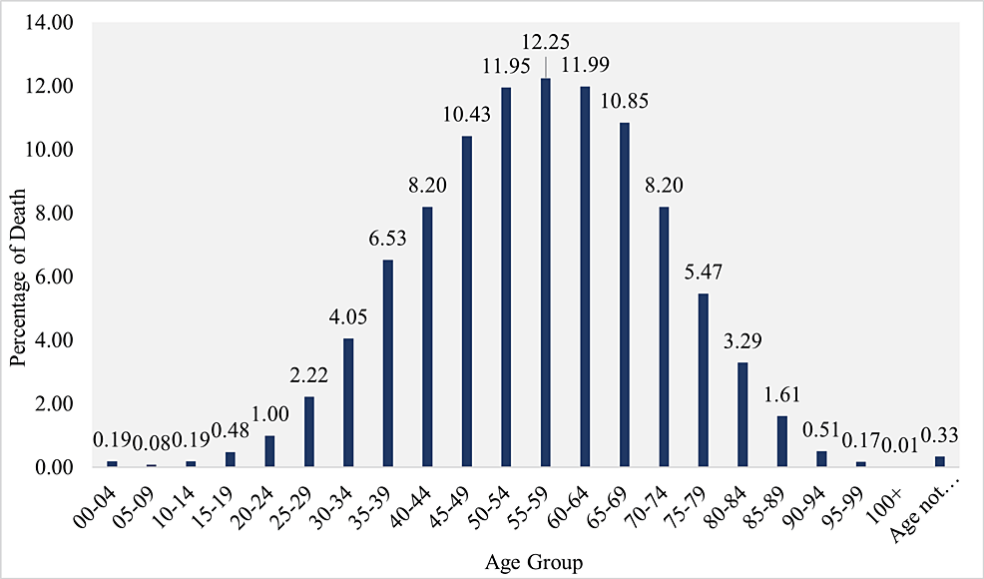
Age distribution of death (in percentage)

**Figure 4:**
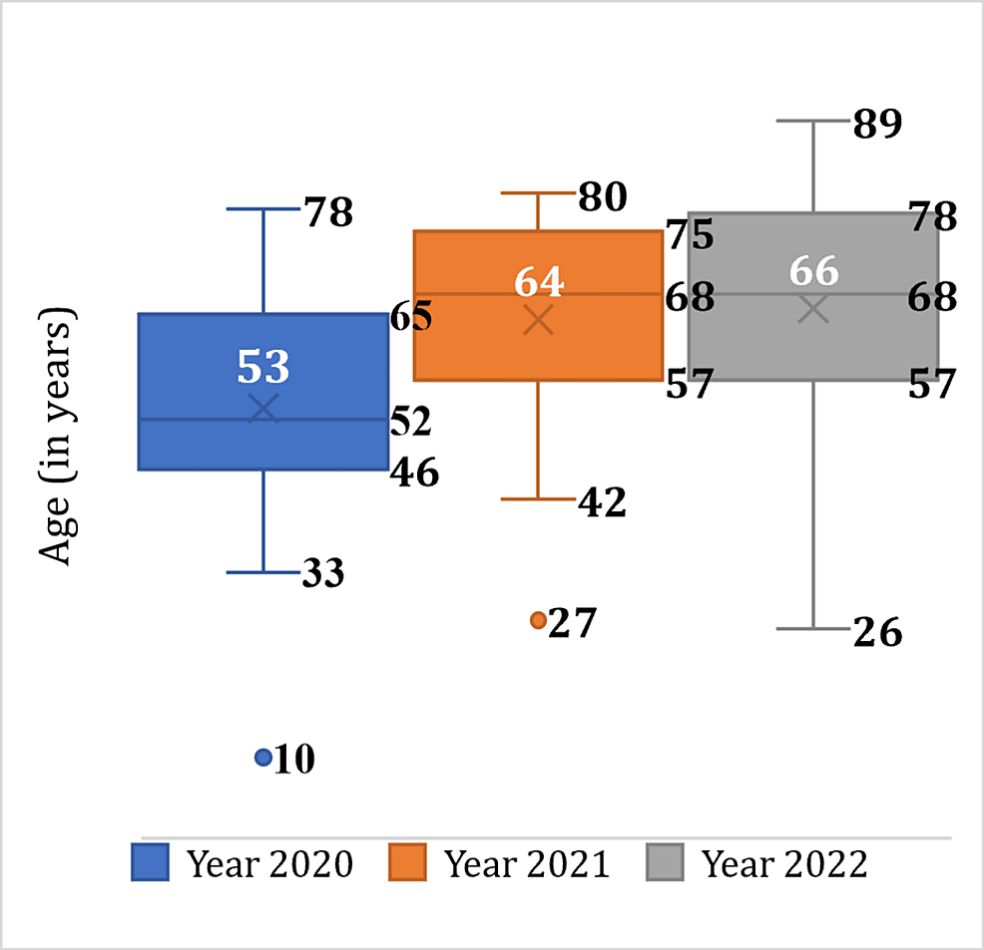
Box plot for age

**Figure 5:**
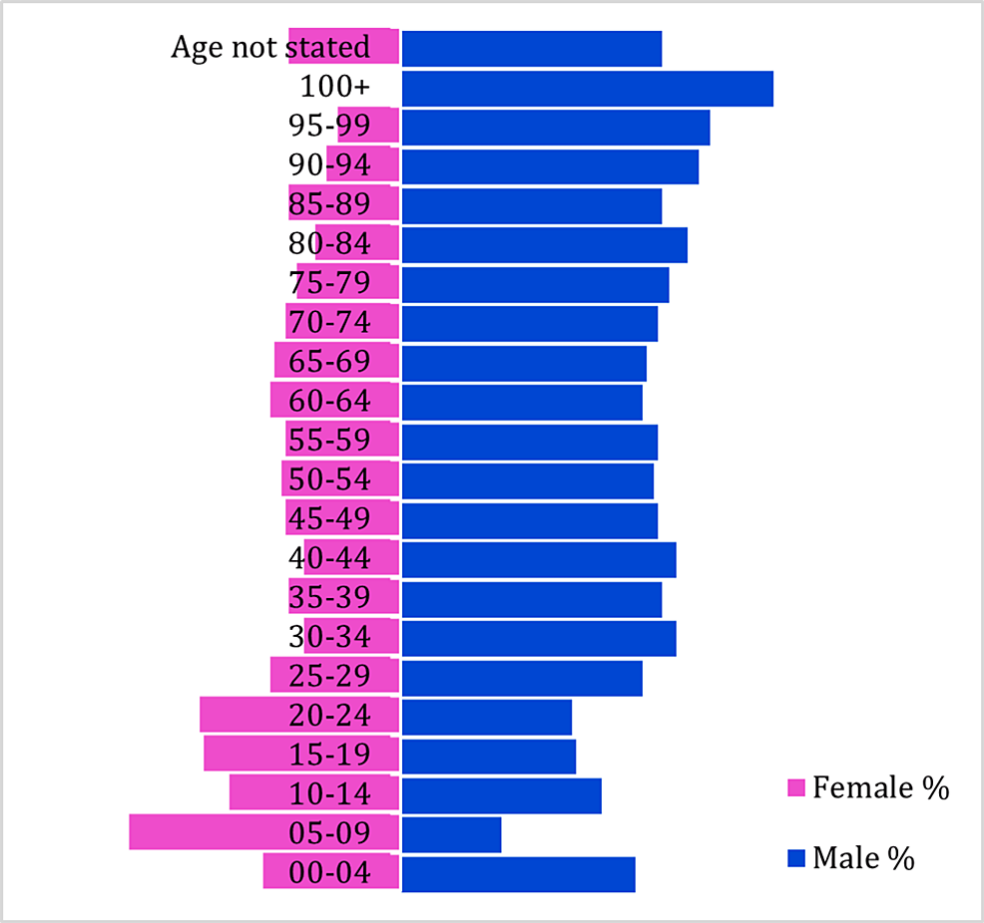
Age pyramid

The research findings revealed significant variations in the prevalence of COVID-19 across different age groups in Chhattisgarh. The highest prevalence was observed among individuals aged 55 to 59, while children (0-9 years) and adolescents (10-19 years) had the lowest prevalence rates (Figure 3). The mean age among the deceased individuals was 55.44 years, with a standard deviation of 15.14 years, indicating that a significant proportion of deaths occurred in older age groups (Figures 4, 5).

**Figure 6:**
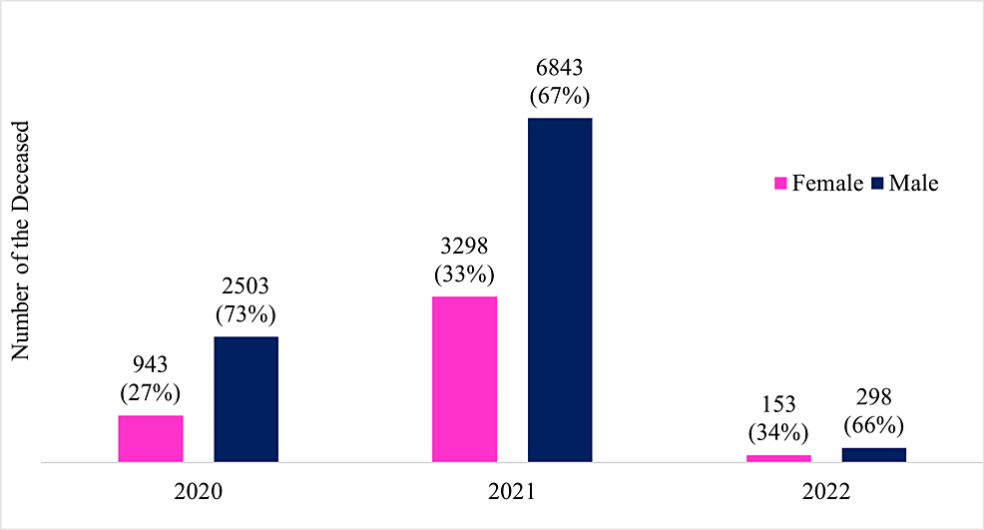
Sex distribution of the deceased (year-wise)

Regarding gender distribution, the pandemic had a more adverse impact on males than females in Chhattisgarh. The ratio of male-to-female deaths was approximately two to one, with 69% of males and 31% of females succumbing to the pandemic (Figure 6). This gender disparity highlights the need for further investigation into the factors influencing the higher vulnerability of males to severe outcomes of COVID-19 in the state.

**Table 1:** Distribution of COVID-19 mortality in Chhattisgarh from March 2020 to March 2022 * Date of admission not present (676)/ brought dead/ transit death/ home death (347) HDU - high-dependency unit

Variable	2020	2021	2022	n=14038 (%)	p-value
	n=3446 (%)	n=10141 (%)	n=451 (%)		
Deaths	3446 (24.5)	10141 (72.2)	451 (3.2)	14038 (100)	
Age					
Less than 18	34 (1.0)	44 (0.4)	11 (2.4)	89 (0.6)	0
18-44	511 (14.8)	2543 (25.1)	78 (17.3)	3132 (22.3)	
45-64	1604 (46.6)	4780 (47.2)	159 (35.3)	6543 (46.6)	
65 and above	1281 (37.1)	2743 (27.0)	203 (45.0)	4227 (30.1)	
Not mentioned	16 (0.5)	31 (0.3)	0 (0.0)	47 (0.3)	
Sex					
Male	2503 (72.6)	6843 (67.5)	298 (66.1)	9644 (68.7)	0
Female	943 (27.4)	3298 (32.5)	153 (33.9)	4394 (31.3)	
Place of residence					
Urban	2383 (69.2)	7111 (70.1)	290 (64.3)	9784 (69.7)	0.023
Rural	1063 (30.8)	3030 (29.9)	161 (35.7)	4254 (30.3)	
Place of death					
Government	2373 (68.9)	6157 (60.7)	231 (51.2)	8761 (62.4)	0
Private facility	973 (28.2)	3737 (36.9)	220 (48.8)	4930 (35.1)	
Home death	61 (1.8)	143 (1.4)	0 (0.0)	204 (1.5)	
Transit death	39 (1.1)	104 (1.0)	0 (0.0)	143 (1.0)	
Type of bed					
ICU/HDU	3018 (87.6)	9221 (90.9)	268 (59.4)	12507 (89.1)	0
Normal	428 (12.4)	920 (9.1)	183 (40.6)	1531 (10.9)	
Comorbidity					
Absent	1199 (34.8)	5845 (57.6)	108 (23.9)	7152 (50.9)	0
Present	2247 (65.2)	4296 (42.4)	343 (76.1)	6886 (49.1)	
Symptoms					
Present	2818 (81.8)	9006 (88.8)	451 (100.0)	12275 (87.4)	0
Absent	628 (18.2)	1135 (11.2)	0 (0.0)	1763 (12.6)	
Vaccine status					
Yes: both doses + first dose	0 (0.0)	8 (0.07) + 3 (0.03)	122 (27.0) + 37 (8.2)	130 (0.9) + 40 (0.3)	0
No					
Vaccine not available	3446 (100)	0 (0.0)	0 (0.0)	3446 (24.5)	
Not received	0 (0.0)	30 (0.3)	292 (64.8)	322 (2.3)	
Data not available	0 (0.0)	10100 (99.6)	0 (0.0)	10100 (71.9)	
Hospitalization days					
Less than 24 hours	532 (15.4)	1515 (14.9)	56 (12.4)	2103 (15.0)	0
24-72 hours	1045 (30.3)	3453 (34.0)	205 (45.5)	4703 (33.5)	
72 hours-1 weeks	587 (17.0)	2248 (22.2)	114 (25.3)	2949 (21.0)	
1 week-1 month	634 (18.4)	2453 (24.2)	75 (16.6)	3162 (22.5)	
>1 month	49 (1.4)	48 (0.5)	1 (0.2)	98 (0.7)	
Data not available*	599 (17.4)	424 (4.2)	0 (0.0)	1023 (7.3)	

The research findings reveal a notable disparity in COVID-19 deaths, with the urban region bearing the highest burden, accounting for 69.7% of the total deaths consistently over the three consecutive years (69.2% in 2020, 70.1% in 2021, and 64.3% in 2022).

Symptomatic COVID-19 cases constituted a significant portion, with 87.4% of patients developing symptoms during the disease course, while approximately 50% of the deceased had underlying comorbidities. Government facilities played a crucial role in handling COVID-19 cases, as 62.4% of the deaths occurred in these settings, whereas private facilities accounted for 35.1%. Notably, 1.5% of deaths occurred at home, and 1% transpired during patient referral or transit (Table 1).

The severity of cases was evident, with 89.1% of the deceased being in intensive care units or high dependency units (ICU/HDU). The data also revealed that 33.5% of COVID-19 patients succumbed to the disease within three days, with a median hospitalization duration of 3±0.06 days. Shockingly, approximately 15% of the patients passed away within 24 hours of hospitalization, while 21% and 22.5% died within one week and one month, respectively. A small fraction (0.7%) of the deceased had prolonged hospitalization, exceeding one month (Table 1).

These findings underscore the urgent need for focused efforts in urban areas to curb the pandemic's impact and enhance healthcare facilities to address the severe cases adequately. Moreover, the high prevalence of comorbidities among the deceased necessitates increased attention to managing such conditions during COVID-19 treatment. Improving timely access to medical care, especially in government facilities, could reduce the mortality rate. The high number of ICU/HDU admissions also calls for bolstering the healthcare system's critical care resources and infrastructure. Addressing these aspects could contribute to better outcomes and reduced fatalities in Chhattisgarh.

## Discussion

The research findings provide valuable insights into the patterns of COVID-19 deaths in Chhattisgarh over two years, shedding light on critical aspects such as geographic distribution, age-related vulnerability, and gender disparities.

The mean age of the deceased individuals (55.44 years) in this study aligns with the findings of a systematic review and meta-analysis conducted by Biswas M et al. on a global scale in 2021 [3]. These collective findings from various studies worldwide consistently indicate a higher mortality risk among the elderly population [4-15]. In line with these global trends, the present study also reveals a higher frequency of deaths among the elderly population in Chhattisgarh. Specifically, individuals above 45 and those under 18 accounted for the highest (80%) and lowest (1%) proportions of COVID-19 mortality in the state, respectively.

Furthermore, our study highlights a noteworthy gender disparity in COVID-19 mortality in Chhattisgarh. Males were disproportionately affected, with a ratio of approximately one female per two male deaths. This finding underscores the need for further investigation into the factors that make males more susceptible to severe outcomes of COVID-19 in the region. Many authors suggest that men are more likely to be infected with COVID-19, especially after 50 years [3, 6, 7, 10-13]. Many previous studies have reported and explored male-female differences in infectious diseases. Many studies worldwide conclude that male deaths were more than females [4-15].

The geographic distribution of COVID-19 deaths reveals a significant concentration of cases in the urban regions of Chhattisgarh, contributing to nearly 70% of the total deaths consistently over the three years of the study. Contributing factors include living conditions, such as high population density, crowded living, and at-risk occupations in urban areas, facilitating disease spread [16]. Urban residents' higher prevalence of comorbidities like obesity and hypertension makes them more vulnerable to severe COVID-19 outcomes. Though rural health infrastructure has improved, it must be enhanced to combat the pandemic's challenges, exacerbated by limited testing rates. Political prioritization of urban regions leaves rural communities with insufficient healthcare support. Targeted interventions, improved infrastructure, increased testing, and public health awareness are crucial for equitable healthcare and reducing COVID-19 mortality across the state [17].

The study provides crucial insights into the distribution of COVID-19 deaths across different healthcare settings in Chhattisgarh, focusing on government hospitals. Our findings demonstrate that government hospitals significantly handled COVID-19 cases, accounting for the highest proportion (62%) of deaths among the study population (n=14038). This aligns with the observations made by Garg et al. (2022), who reported that government hospitals were responsible for 69% of hospitalizations for COVID-19 treatment in Chhattisgarh [18].

Remarkably, a substantial proportion of deaths occurred shortly after hospitalization. Approximately 33% of the total COVID-19 deaths in our study (n=14038) and 37% in a subset of patients (n=356) transpired within three days of admission. Moreover, 15% of patients succumbed to the disease within 24 hours of hospitalization. These findings echo patterns seen in other states of India, such as Delhi, Bengaluru, and Madhya Pradesh, where early fatalities after hospital admission have been observed [19-21]. Supporting evidence from a study by Prasad et al. (2022) in a tertiary care hospital in Chhattisgarh indicates that nearly 46% of non-survivors passed away within five days of admission [22]. These findings collectively underscore the critical importance of early detection, prompt medical attention, and appropriate management protocols for COVID-19 patients, especially during the initial days of hospitalization.

The higher percentage of deaths occurring in government hospitals necessitates a comprehensive evaluation of these settings' healthcare infrastructure and resources. Improving hospital preparedness, ensuring adequate staffing, and enhancing the availability of medical equipment and critical care resources may help to minimize COVID-19 fatalities in these healthcare facilities. Enhancing public awareness and healthcare-seeking behavior could lead to earlier hospital admissions and improved patient outcomes. By addressing these factors, Chhattisgarh can better equip itself to handle the COVID-19 pandemic and optimize patient care, ultimately contributing to reduced mortality rates and better overall pandemic management.

Limitations

The study's primary limitation lies in its reliance on retrospective data, which may be subject to incomplete or inaccurate recording. Additionally, the study's scope is confined to the information available in the death records, limiting the exploration of potential confounding factors. Despite these limitations, this research serves as an essential step in understanding mortality patterns in Chhattisgarh and can provide valuable insights for public health interventions and policies.

## Conclusions

Raipur, Durg, and Bilaspur (urban-centric) were the most affected districts throughout the pandemic. Male predominance was seen in the study; around 70 % of the patients were male. Almost all (12,275; 87.4%) COVID-19-positive patients were symptomatic in the study group. Nearly all three waves were observed second wave was the deadliest, especially for the elders.
